# BG34-200 Immunotherapy of Advanced Melanoma

**DOI:** 10.3390/cancers14235911

**Published:** 2022-11-30

**Authors:** Veronique Roche, Victor Sandoval, Zachary Senders, Joshua Lyons, Claire Wolford, Mei Zhang

**Affiliations:** 1 Department of Biomedical Engineering, Case Western Reserve University School of Medicine, 11100 Euclid Avenue, Cleveland, OH 44106, USA; 2 Case Comprehensive Cancer Center, Case Western Reserve University, Cleveland, OH 44106, USA; 3 University Hospitals Cleveland Medical Center, Case Western Reserve University, 11100 Euclid Avenue, Cleveland, OH 44016, USA

**Keywords:** advanced melanoma, immunotherapy, carbohydrate, innate immunity, tumor-draining lymph nodes, combination therapy, T-cell therapy

## Abstract

**Simple Summary:**

Immunotherapy using immune checkpoint inhibitors is now the first line standard of care for advanced melanoma but >50% of patients still do not benefit. Using a recently developed plant-derived glucan molecule called BG34-200, we found that targeting elements of the myeloid components are useful in creating systemic antitumor immune responses in advanced melanoma. Scientific findings from this study suggest that BG34-200-based combination therapy can be developed to benefit patients with advanced melanoma who do not respond to current standard of care therapies with and without immunotherapy.

**Abstract:**

High levels of myeloid-derived cells are characteristic of the tumor microenvironment (TME) of advanced melanoma. These cells interact with tumor cells to suppress the development of antitumor immune responses, regulate tumor metastasis, and drive cancer’s resistance to virtually all types of therapy. Therefore, methods to disrupt tumor-associated myeloid cell function are actively being sought to find a cure. Our team has recently developed a plant-derived carbohydrate molecule, BG34-200, that modulates tumor-associated myeloid cells by targeting the cell surface receptor CD11b. In this study, we found that BG34-200 IV administration could significantly inhibit tumor growth and improve survival in B16F10 mice with advanced melanoma. Our data supported a model that the entry of BG34-200 into circulating melanoma tumor-associated inflammatory monocytes (TAIMs) could trigger a sequential immune activation: the BG34-200^+^ TAIM subsets migrated to tumor and differentiated into monocyte-derived dendritic cells (mo-DCs); then, the BG34-200^+^ mo-DCs migrated to tumor draining lymph nodes, where they triggered the generation of tumor-antigen-specific T cells. Based upon these results, we combined BG34-200 treatment with adoptive transfer of TdLN-derived T cells to treat advanced melanoma, which significantly improved animal survival and helped tumor-free survivors be resistant to a second tumor-cell challenge. The scientific findings from this study will allow us to develop new technology and apply BG34-200-based immunotherapy to patients with advanced melanoma who have not responded to current standard of care therapies with and without immunotherapy.

## 1. Introduction

Advanced melanoma refers to melanoma that has spread from its primary sites to draining lymph nodes and other parts of the body. The average life expectancy for advanced melanoma patient is ~6–22 months [1,2,3]. Recent progress in immune-mediated approaches, including immune checkpoint blockade therapy, have resulted in new hope for advanced cancers. Despite the initial success, a considerable number of patients with advanced disease do not respond to this approach [4].

Increasing evidence has shown that the melanoma tumor microenvironment (TME) consists of heterogeneous malignant cells located in a complex environment [5]. The limitation of immunotherapy is mainly due to gaps in knowledge about how TME suppresses host immunity and how to overcome this challenge. Recent clinical and experimental results suggest that myeloid cell activation in the TME can suppress host antitumor immune responses, promote tumor metastasis, and drive resistance to virtually all types of therapy [6,7]. Thus, methods to disrupt tumor-associated myeloid cell function are actively being sought to overcome challenges in immunotherapy to find a cure.

Our team has recently developed a new and safe carbohydrate molecule that is isolated from oat bran and possesses an unusual capacity to modulate myeloid-derived cells [8,9,10]. Our technology discovery was that the oat-derived β-(1,3)-(1,4)-glucans (BG34) with an average molecular weight (*M*w) of ~200 kDa (BG34-200) could induce antitumor effects by modulating myeloid cells via interacting with cell surface marker CD11b (CR3; CD11b/CD18; Mac-1). Although BG34 of other molecular sizes could bind to CD11b, the binding strengths were different, and only BG34-200 could induce beneficial biological responses. Importantly, our data suggest that the extended carbohydrate polymer chain of BG34-200 contains sufficient copies of binding units that formed multivalent engagement with CD11b lectin binding sites. The concomitant clustering of the CD11b lectin binding sites and the carbohydrate recognition units mediated a biologically relevant binding affinity that effectively triggered CD11b-mediated biological responses in the context of disease [8,9,10].

CD11b is a C-type lectin/integrin that plays an important role in host immune recognition. CD11b can sense and respond to pathogen-associated molecular pattern (PAMP) carbohydrate ligands to trigger immediate innate immune recognition and activation [11,12]. PAMP molecules are highly conserved carbohydrate structures uniquely found in bacteria, viral, plant, and fungal pathogens but not in the hosts, making them promising candidates to be used to modulate CD11b^+^ myeloid cells in disease conditions. However, CD11b-based immune recognition of carbohydrate ligands is an intricate biological process greatly affected by the geometry, number, and type of sugars [13,14,15]. This has created a major challenge to understand the molecular basis by which CD11b converts differences in receptor–ligand binding into subsequent immune signaling responses and to use this knowledge for therapeutic development. Our published results showed that BG34-200 with a defined structure and molecular size can trigger a direct, specific, and multivalent binding to specific epitopes of CD11b [8,9,10]. The peptide epitopes are distinct from those previously used to develop CD11b-targeted antibodies or agents [14,16].

In this study, we investigated the immune activation induced by the BG34-200-CD11b engagement in the context of advanced melanoma and used this knowledge to develop BG34-200 combinational immunotherapy. Our data demonstrate that targeting elements of the myeloid components are useful in creating systemic antitumor immune responses in advanced disease. Our findings suggest that the BG34-200 therapy can reduce risk in pre-invasive disease, be used as an adjuvant for standard therapies, or synergized with cellular immunotherapy for advanced and aggressive melanoma.

## 2. Materials and Methods

### 2.1. Cell Culture

Mouse models of melanoma were developed using B16F10 (CRL6475TM) cell lines purchased from ATCC (ATCC, Manassas, VA, USA). The cells were cultured for 3–4 passages at maximum 80% confluency in 10% fetal bovine serum (Invitrogen, Grand Island, NY, USA) in DMEM media and 1% penicillin/streptomycin (ATCC, Manassas, VA, USA) in a T175 flask under standard conditions (37 °C, 5% CO_2_, 95% humidity) and tested regularly in-house for mycoplasma.

### 2.2. Animals and Tumor Models

CD11b-KO (B6.129S4-Itgamt^m1Myd^/J), CCR2-KO (B6.129S4-*Ccr2^tm1Ifc^*/J) CCR7-KO (B6.129P2(C)-*Ccr7^tm1Rfor^*/J), CD11c-Venus (B6.Cg-Tg(Itgax-Venus)1Mnz/J), and LysM-GFP ((B6.129P2-*Lyz2^tm1(cre)Ifo^*/J) mice were purchased from the Jackson Laboratory (Bar Harbor, ME, USA). CD11b-KO animals have a diminished ability to clear thioglycollate-induced neutrophils and have reduced mast cell numbers in the dorsal skin and peritoneal wall/cavity. Neutrophils from these animals are deficient in spreading, phagocytosing complement-opsonized particles, and in several Fc-mediated functions. CCR2-KO animals display an absence of the CCR2 chemokine receptor which affects monocyte/macrophage infiltration leading to inflammatory responses, which, in turn, impacts various systematic processes, including immune responses to various pathogens. CCR7-KO animals show delayed primary B- or T-cell immune responses. Lymph nodes from homozygous mice are devoid of naive T cells and dendritic cells (DCs), but the T-cell populations in the blood, the red pulp of the spleen, and in the bone marrow are greatly expanded. Secondary lymph organs exhibit morphological abnormalities, and adoptive transfer experiments demonstrate impaired B- and T-cell migration. CD11c-Venus animals express yellow fluorescent protein (YFP) under the transcriptional control of the mouse integrin alpha X (Cd11c) promoter, which is exclusively expressed in dendritic cells of the immune system. LysM-GFP animals have a LysMcre knock-in/knock-out allele that has a nuclear-localized Cre recombinase inserted into the first coding ATG of the lysozyme 2 gene (*Lyz2*); thus, both abolishing endogenous *Lyz2* gene function and placing NLS-Cre expression under the control of the endogenous *Lyz2* promoter/enhancer elements were useful to study myeloid cell lineages such as monocytes, mature macrophages, and granulocytes. Mice were acclimatized to their new pathogen-free animal facility for at least 2 days before starting experiments. For the advanced B16F10 model, 1.5 million tumor cells were subcutaneously injected into the left flank of wild-type C57BL/6J mice on day 0. Previous studies, including ours, have shown that subcutaneous inoculation of 1 to 1.5 million of B16F10 cells in immunocompetent C57BL/6J mice resulted in efficient kinetic tumor growth with a doubling time of approximately 2 days, produced solid tumors within 2–3 weeks, and generated metastasis with a 100% frequency. Mice can be studied for ~22 days before reaching the euthanasia criteria of tumor burden. Tumor scabbing and ulcerations are common clinical symptoms associated with subcutaneous and intradermal B16-F10 tumor growth. Tumor monitoring was performed with a caliper, and the volume calculation was based on the following formula: V = (4/3)π (L/2) x (w/2) x (h/2). In this study, treatment or control was IV-administered on day 4. PBS served as control. BG34-200 and BG34-200-AF647 samples were prepared, synthesized, and characterized according to our published protocols [15,16]. For the mice that were used to collect blood, tumor, and TdLN samples, the mice were sacrificed at 10 h, 24 h, and 72 h, respectively, after IV injection of treatment or control to collect the corresponding samples. In the T-cell transfer in vivo study, TdLNs were harvested from donor B16F10 mice receiving a PBS (group I) or BG34-200 (groups II and III) intravenous injection, cultured/expanded using T-cell activation beads (CD3/CD28) in the presence of recombinant IL2 cytokines, and adoptively transferred to recipient mice. For the donor mice, one million B16F10 cells were injected into left flanks on day 0; PBS (group I) or BG34-200 (groups II and III) were intravenously administrated on day 4. For the recipient mice, 1.5 million B16F10 cells were injected into left flanks on day 0; PBS (groups I and II) or BG34-200 (group III) were intravenously administrated on day 4. Adoptive transfer of T cells was conducted on day 6. Age- (6 to 12 weeks) and sex-matched mice were used for all experiments. All mice were maintained under specific pathogen-free conditions in the Animal Research Center of Case Western Reserve University (CWRU). All animal protocols were approved by the Institutional Animal Care and Use Committee (IACUC) of CWRU Animal Research Center.

### 2.3. Fluorescence Imaging

Non-invasive whole-body fluorescence imaging was carried out using IVIS Spectrum (Perkin Elmer, Waltham, MA, USA). All mice were anesthetized via isoflurane inhalation (2% isoflurane, oxygen flow rate of 2 L/min) using the XGI-8 Gas Anesthesia System prior to and during imaging. The mice were imaged in both dorsal and flank positions at indicated days’ post infection. Images were acquired and analyzed with the manufacturer’s Living Image v4.7.3 in vivo software package. Standard regions of interest (ROIs) were drawn around tumor locations. ROI values were reported as radiant efficiency [(p/sec/cm2/sr)/(µW/cm2)].

### 2.4. Preparation of Plasma and Cell Samples

For the preparation of plasma samples, mouse blood was drawn into purple-top, EDTA-coated BD vacutainer tubes (BD, Franklin Lakes, NJ, USA) according to the following protocol: Completely fill the Vacutainer with pooled blood whenever possible to eliminate dilution from the anticoagulant or preservative. Immediately mix the blood by gently and thoroughly inverting the tube five to ten times. Separate plasma by centrifugation (15 min at 2500 rpm) and store samples at −80^o^C. For the preparation of white blood cells (WBCs), mouse blood was drawn into purple-top, EDTA-coated BD vacutainer tubes and processed according to the following protocol: Centrifuge the whole blood at 300 g for 5 min, discard the plasma layer, and dilute the remaining cells with RBC lysis buffer at a ratio recommended by the manufacturer (ThermoFisher Scientific, Waltham, MA, USA). The RBC lysed blood cell suspension was processed to collect WBC according to published protocol [17]. For the preparation of digested tumor cells, the tumor samples were collected and placed into cold complete medium and processed immediately. Tumor tissue was dissociated for 4–6 h at 37 °C using a cocktail of collagenase III, DNase I, and trypsin. Cell suspensions were washed, resuspended, and lysed by RBC lysis buffer. For the preparation of TdLN cells, the lymph nodes were washed three times using complete medium, gently pulled apart to release cells, and resuspended in stain buffer.

### 2.5. Surface Stain of Cells

Single-cell suspensions were first blocked for 15min at room temperature in a PBS-2% FBS solution, then stained for surface molecules for 30 min at room temperature. After staining, cells were washed by wash buffer twice and centrifuged to collect cell pellets. The pellets were then fixed in 20 volumes of fixation buffer (BD Bioscience, San Jose, CA, USA).

### 2.6. Histology Staining

The frequency of tumor-infiltrating T cells (TILs) of B16F10 mice receiving PBS or BG4-200 IV administration was determined by immunostaining of fixed tumor tissues. Fixed and embedded sections were stained using an anti-CD3 monoclonal antibody. Evaluation between both conditions was done visually by comparing the overall brown surface (CD3^+^ cells) compared to the counter-stain (purple cells).

### 2.7. Fluorescence-Activated Cell Sorting and Analysis (FACS)

Fluorochrome-conjugated antibodies for mouse MHC II (M5/114.15.2) and MHC I (28-14-8) were purchased from ThermoFisher Scientific (ThermoFisher Scientific, Waltham, MA, USA). All the other antibodies were purchased from BD Biosciences (BD Biosciences, San Jose, CA, USA). The clones are: CD3 (145-2C11), CD19 (1D3), CD20 (GOT214A), CD56 (809220), CD80 (1G10/B7), CD62L (MEL-14), CD86 (PO3), CD4 (RM4-5), CD8 (5H10-1), CD25 (7D4), CD44 (IM7), CD11c (HL3), CD40 (3/23), CD11b (M1/70), CD11c (HL3), CD54 (3E2), CD83 (Michel-19), CCR2 (475301), Ly6C (AL-21), and Ly6G (1A8). T-cell proliferation was measured using e-Flor 660-Ki67 proliferation assay (Thermofisher) according to the manufacturer’s protocol. The stained and fixed cells were loaded for FACS analysis or sorting. For the single and multicolor FACS, BD LSR II (BD Biosciences) was used for data acquisition. The data were analyzed using Winlist 10.0. For cell sorting, and the fixed cells were sorted according to their surface marker on a BD FACS Aria^TM^ III cell sorter (BD Biosciences).

### 2.8. Total RNA Isolation and Quantitative Real-Time PCR

Total RNA was isolated from fresh cells using the RNeasy Plus Mini Kit (Qiagen, Valencia, CA, USA) according to the manufacturer’s protocol. Between column washes, an on-column DNase digestion was performed using the RNase-free DNase kit (Qiagen). Total RNA was isolated from fixed cells using reagents from the RNeasy FFPE kit and the RNeasy Plus Mini Kit (Qiagen) according to the manufacturer’s protocol. Assessment of RNA quality was performed using the Agilent 2100 bioanalyzer (Agilent Technologies, Santa Clara, CA, USA). Real-time PCR was performed with a SYBR Green real-time PCR kit (ThermoFisher Scientific, Waltham, MA, USA) and LightCycler 1.5 instrument (Roche Life Sciences, Pleasanton, CA, USA). Primers purchased from Origene (Origene, San Francisco, CA, USA) are: PU.1_ F: 5′-GGGAGAGCCATAGCGACCAT-3′ R:5′-TCTTGGCCACCAGGTCTCCTA-3′; MafB.1_5′-TACTGGATGGCGAGCAACTACC-3′ R: 5′-GAGCTTCGACGGCTTCCGTAGT-3′; CD80_F: 5′-CCTCAAGTTTCCATGTCCAAGGC-3′, R: 5′-GAGGAGAGTTGTAACGGCAAGG-3′; CD83_F: 5′-ACCGTGGTTCTGAAGGTGACAG-3′, R: 3′-CCAGAGAGAAGAGCAACACAGC-3′; CD86_F: 5′-ACGTATTGGAAGGAGATTACAGCT-3′, R:5′- TCTGTCAGCGTTACTATCCCGC-3′; CD40_F: 5′-ACCAGCAAGGATTGCGAGGCAT-3′, R:5′-GGATGACAGACGGTATCAGTGG-3′; and OX40L_F:5′-GGAAGAAGACGCTAAGGCTGGT-3′, R:5′-CTGGTAACTGCTCCTCTGAGTC-3′; GP100_ F:5′-ATTGCTCTGCTTATCGGCTGCT-3′, R: 5′-CACCATTCCTCCAATATCCCTCT-3′; DCT_F: 5′-AGCAGTATGGCTGGAGCACT-3′, R: 5′-AGCCCTTTCC-TCTCCTCTCA-3′; GAPDH_ F: 5′- CAAGGTCATCCATGACAACTTTG-3′, R: 5′-GTCCACCACCCTGTTGCTGTAG-3′.

### 2.9. Adoptive T-Cell Transfer

The TdLN-derived T-cell culture and expansion was conducted based on our published protocols [18,19,20]. Briefly, one million B16F10 cells were subcutaneously injected into the flanks of donor WT C57BL/6J mice on day 0. PBS or BG34-200 solution (100 mg/kg) were IV injected on day 4. Mice were sacrificed on day 6 to collect TdLN samples. Freshly collected TdLN cells were cultured at one million cells per well in a 24-well plate (2 mL complete medium/well). Anti-CD3/anti-CD28 beads (Dynal AS, Oslo, Norway) were added according to manufacturer’s instructions. IL-2 (100 U/mL) (Invitrogen) was added to the culture. Cells were split to a concentration of 0.25 × 10^6^ cells/mL by adding fresh medium and then IL-2 every other day until the end of the culture. Cells were cultured in a humidified 5% CO_2_ incubator at 37 °C. Cells that contained over 65% CD3^+^ T cells were adoptively transferred to recipient mice at 1 million cells per mouse.

### 2.10. Statistics

The number of cell cultures and animals used for the experiments are reported in the graphs of the figures and in the figure legends. Statistical analysis was performed using GraphPad Prism 9.0 (GraphPad Software Inc., San Diego, CA, USA). Although no statistical methods were used to predetermine sample sizes, our sample sizes were similar to those generally employed in the field. For comparisons of multiple groups, one-way ANOVA followed by Holm–Sidak multiple comparison tests were used. In vitro comparison was performed with two-tailed Student’s *t* test. Comparison of survival curves was performed by the Log-rank (Mantel–Cox) test. All statistical analyses were performed using GraphPad Prism 9.0 (GraphPad Inc.). Significance was set at *p* < 0.05.

## 3. Results

### 3.1. BG34-200 IV Administration Induces Antitumor Effects and Reduces Lymph Node Metastases in B16F10 Model of Advanced Melanoma

**Figure 1:**
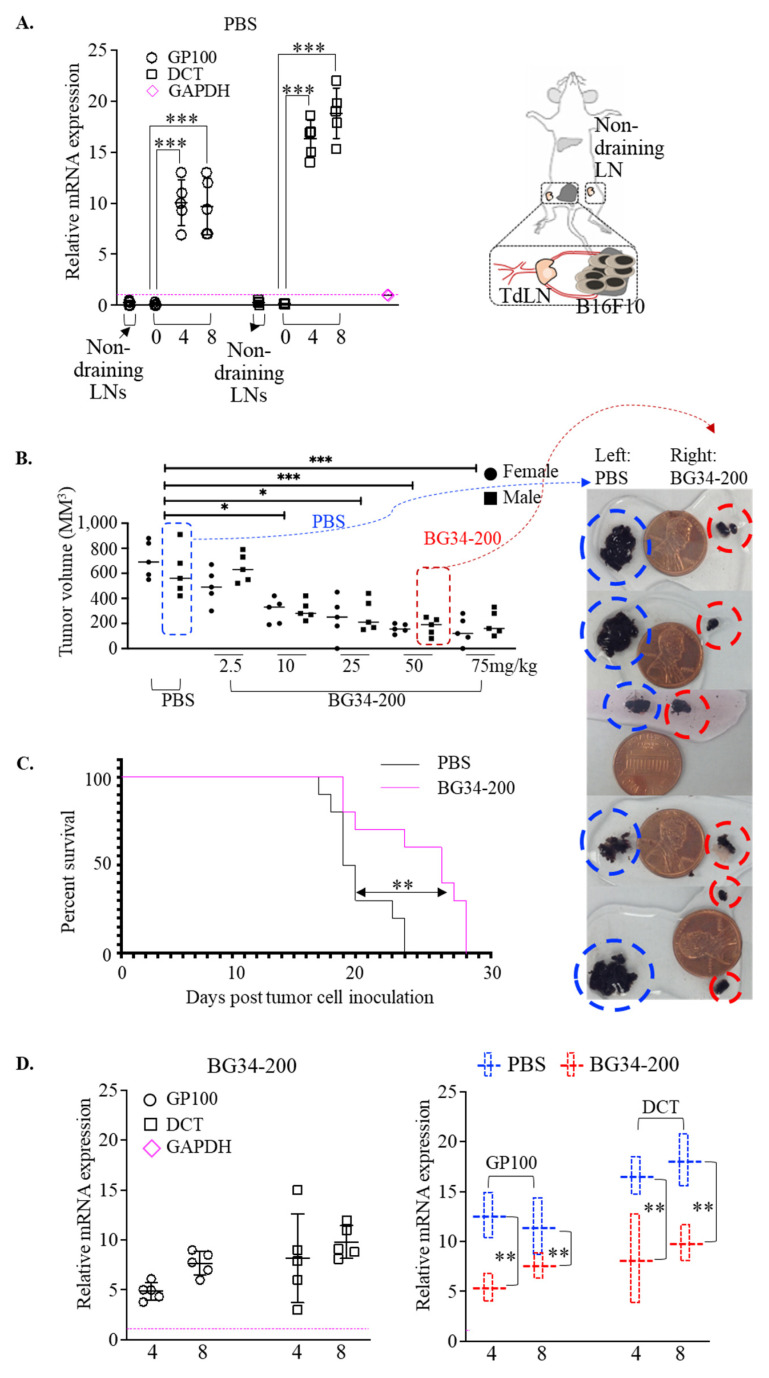
BG34-200 IV administration induces antitumor effect in B16F10 model of advanced melanoma. (**A**). Relative mRNA expression of GP100 and DCT in lymph nodes (LNs) of control B16F10 mice (PBS-treated) as determined by RT-PCR. One and a half million B16F10 cells were inoculated into the left flank of C57BL/6J mice on day 0. Tumor-draining lymph nodes (TdLNs) were harvested on day 0, 4, and 8 to examine the mRNA expression. The non-draining LNs harvested on day 0 serve as control. The mRNA expression level was normalized to internal control GAPDH. (**B**). Tumor volumes in mice receiving IV administration of PBS or BG34-200 solution. PBS or BG34-200 of 5 different doses was IV-administrated on days 4 and 10 after tumor cell inoculation. Mice were euthanized on day 18 to measure the tumor volume. *n* = 10/group. Equal number of female and male mice were used. (**C**). Kaplan–Meier overall survival curves of B16F10 mice receiving PBS or BG34-200 (50 mg/kg) weekly for 2 weeks. (**D**). Relative mRNA expression of GP100 and DCT in lymph nodes of B16F10 mice treated with BG34-200 (left panel); relative GP100 and DCT mRNA expression comparison of control mice (PBS) and treated mice (BG34-200) in the lymph nodes harvested at 4 and 8 days after inoculation of 1.5 million B16F10 cells into left flank. The mRNA expression level was normalized to internal control GAPDH. For A-D, *, *p* < 0.05; **, *p* < 0.01, ***, *p* < 0.001.

We examined the tumor metastasis window of the B16F10 model by checking the mRNA expression of melanoma-specific genes in the draining lymph nodes (Figure 1A). This is because sentinel lymph nodes are the most common first site of metastases for advanced melanoma [21]. The GP100 gene is expressed in mouse/human melanocytic cells and used as specific marker for melanoma detection [22,23]. The DCT gene is highly expressed in chemo- and radiotherapy-resistant mouse/human melanoma cells that undergo de-differentiation and thus can be used as a marker in complement of GP100 for melanoma detection and staging [24,25,26]. Our data showed that the mRNA of these two genes was significantly upregulated in tumor draining lymph nodes (TdLNs) as early as 4 days after 1.5 million B16F10 cells were inoculated subcutaneously into the left flanks (Figure 1A). These results suggest that the B16F10 model could effectively recapitulate the key aspects of the relevant advanced human melanoma as it allowed tumor growth at the primary site as well as metastatic migration to the sentinel-draining lymph nodes. Using this animal model, we found that BG34-200 IV administration induced antitumor effects in mice with advanced disease in a dose-dependent manner (Figure 1B), significantly improved animal survival (Figure 1C), and reduced lymph node metastases (Figure 1D).

### 3.2. Direct Entry of AF647-Tagged BG34-200 (BG34-200-AF647) to Circulating CD11b^+^ Cells Facilitates Plasma Clearance of the Compound and Affects Compound Migration and Distribution

**Figure 2:**
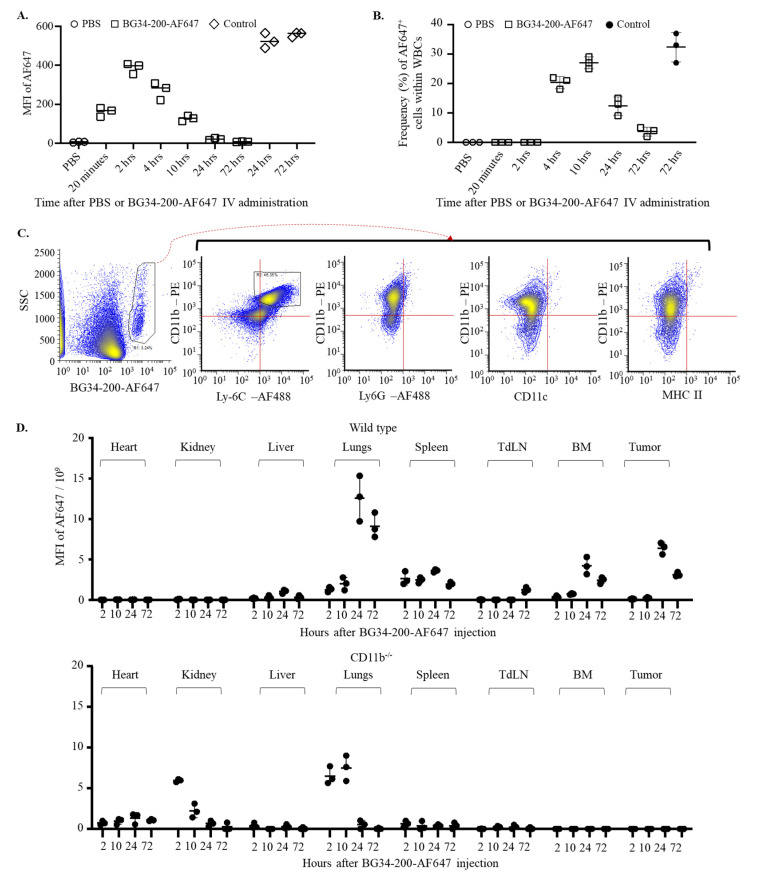
Direct entry of BG34-200-AF647 into circulating CD11b^+^ cells facilitates plasma clearance of the compound and mediates absorption in peripheral organs and tumor. (**A**). Fluorescence signal in plasma samples over 72 h after IV administration of PBS or BG34-200AF647 in B16F10 mice. Tumor-free mouse plasma samples spiked with BG34-200-AF647 for 24 and 72 h served as positive controls. (**B**). Frequency of AF647^+^ WBCs over 72 h after IV administration of BG34-200AF647 or PBS. Tumor-bearing mouse WBCs spiked with BG34-200-AF647 for 72 h served as positive control. (**C**). FACS analysis (phenotyping) of the BG34-200-AF647^+^ WBCs population harvested at 10 hrs after BG34-200-AF647^+^ IV administration using PE-tagged CD11b and AF488-tagged Ly6C, Ly6G, CD11c, or MHC II. (**D**). MFI of BG34-200-Af647 signals in the heart, kidneys, liver, lungs, spleen, tumor-draining lymph nodes (TdLNs), bone marrow (BM), and tumor at 2, 10, 24, and 72 h after IV administration of BG34-200-AF647 compound in the wild-type (WT) mice (top panel) and CD11c−/− mice (bottom panel) as determined by colorimetric analysis. For (**A**,**B**,**D**), *n* = 9; each data point represents a sample pooled from three mice, and each data point was determined in triplicate. Data are graphed as mean ± SD.

Firstly, we examined plasma concentrations of the BG34-200-AF647 compound following IV administration of 50 mg/kg. BG34-200-AF647 was synthesized and characterized in our previous studies and displayed high stability against different pH and various digestive enzymes [8]. Carbohydrate molecules have been shown to be eliminated from plasma within hours to 12 months; plasma half-life is thought to be affected by the chemical structure, molecular size, solubility, and the way of administration [26,27]. Here, our results showed that the complete plasma clearance took around 24 h following BG34-200-AF647 IV administration, as evidenced by the time-dependent decrease in fluorescence signals in plasma samples (Figure 2A).

Next, we examined the frequency of the BG34-200-AF647^+^ circulating cells at different time points after IV administration. The BG34-200-AF647^+^ cells were detectable between 2 and 4 h, increased to reach a peak value at 10 h, and decreased to baseline level at 72 h (Figure 2B). The phenotypic analysis of these cells at 10 hrs after BG34-200-AF647^+^ IV administration showed positive expression of CD11b and Ly6C and negative expression of MHCII, CD11c, and Ly6G, suggesting that they were monocytic cells rather than granulocytic (Figure 2C).

Furthermore, we examined the BG34-200-AF647 concentrations in tumors, various organs, and tissues. The BG34-200-AF647 fluorescent signal was detected in the tumor, lungs, and bone marrow (BM) between 10 and 72 h, and in TdLNs between 24 and 72 h (Figure 2D, top). In contrast, the BG34-200-AF647 signal was not detected in the tumors, BM, and TdLNs in CD11b knockout mice (CD11b^−/−^, B6.129S4-*Itgam^tm1Myd^*/J) (Figure 2D, bottom). Because the CD11b^−/−^ mice lacked CD11b protein expression on neutrophils, monocytes, and monocyte-derived macrophages or dendritic cells, these results suggest that the CD11b^+^ cells played very important role in the migration and distribution of the BG34-200-AF647 compound in vivo. On the other hand, the AF647 signal was detected in the lungs and kidneys of CD11b^−/−^ mice and decreased to baseline levels within 24 h, suggesting that the lungs and kidneys were involved in eliminating the compound in the absence of CD11b^+^ cells. Collectively, these results suggest that the migration and distribution of BG34-200-AF647 are affected by CD11b^+^ monocytic cells.

Of note, we observed a high level of BG34-200-AF647 in the lungs in WT mice at 24 and 72 h. Water soluble polysaccharide (or carbohydrate polymer) concentrations in lung tissues have been correlated with pulmonary pharmacokinetics triggered by the recruitment of macrophages and neutrophils that have taken up these large molecules. Since the concentration–time profiles in lungs are largely influenced by the structure, size, and functional groups of the polysaccharide compounds, there is no standard translational PK model to describe the pulmonary concentration–time profiles. To the best of our knowledge, this is the first report of pulmonary PK of water soluble β-glucan of 200 kDa upon IV administration.

### 3.3. BG34-200-AF647 Migration and Infiltration to B16F10 Tumor Are Dependent on Circulating Cells Expressing Tumor-Associated Inflammatory Monocyte Markers CD11b and CCR2

**Figure 3:**
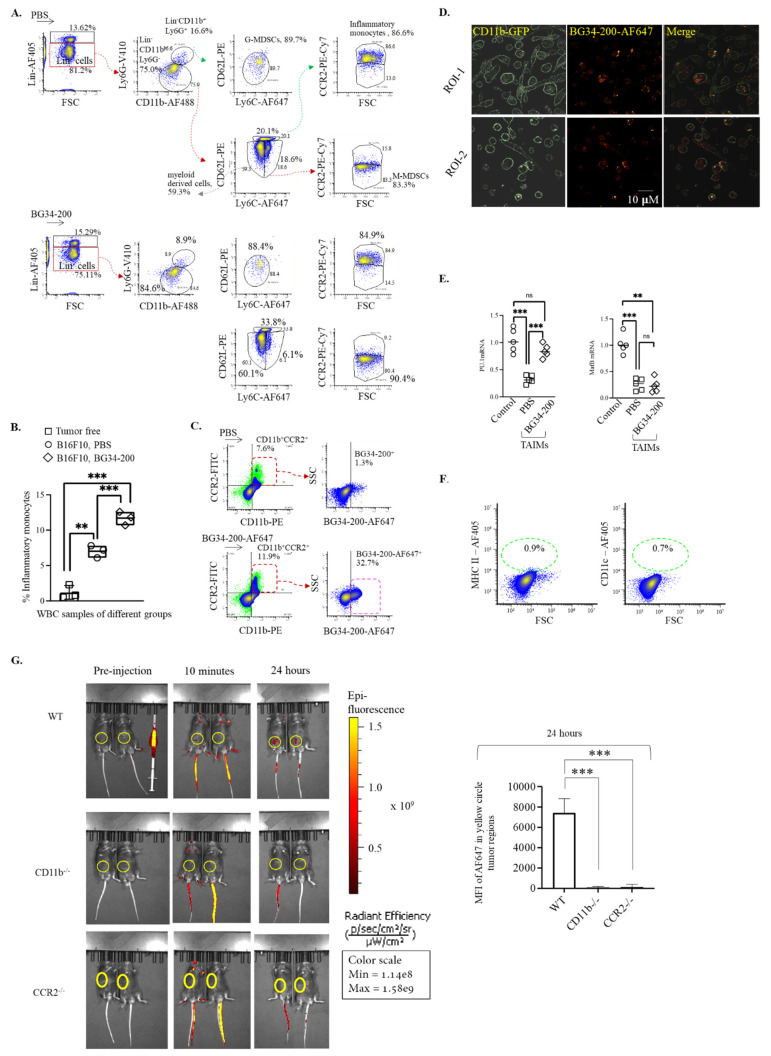
BG34-200 IV administration shows impact on CD11b^+^ cell differentiation. (**A**). Six-color FACS analysis to identify granulocytic MDSCs (G-MDSCs), monocytic MDSCs (M-MDSCs), inflammatory monocytes, and resident monocytes (patrolling monocytes) in the whole blood samples of mice receiving PBS or BG34-200. (**B**). Frequency of inflammatory monocytes within the white blood cells (WBCs) of B16F10 mice receiving PBS or BG34-200 administration. Tumor-free mouse WBC served as control. (**C**). Frequency of BG34-200-AF647^+^ cells in CD11b^+^CCR2^+^ cells as determined by FACS analysis. (**D**). Fluorescence microscopy of the internalization of the BG34-200-AF647 compound by the CD11b-GFP^+^ cells. (**E**). Relative mRNA expression levels of PU.1 (left) and MafB (right) in the TAIMs sorted from PBS- or BG34-200-treated mouse blood as determined by RT-PCR. BM-derived inflammatory monocytes from tumor-free mice served as control. (**F**). Expression of MHC II and CD11c by CD11b^+^CCR2^+^BG34-200-AF647^+^ cells, as determined by FACS analysis. (**G**). Mean fluorescence intensity (MFI) of BG34-200-AF647 in WT, CD11b^−/−^, and CCR2^−/−^ mice pre-injection, 10 min and 24 h post-injection using non-invasive IVIS spectrum imaging. The image of two mice in each group represented one of the two repeats (left). All mice received left flank injection of 1.5 million B16F10 tumor cells 4 days before BG34-200-AF647 injection and exhibited palpable tumors. Tumors were indicated by yellow circles. MFI of BG34-200-AF647 in the yellow circles was quantified. *n* = 4 mice per group. Data presented as mean ± SD. For (**B**,**E**,**G**), **, *p* < 0.01, ***, *p* < 0.001.

Since CD11b is a surface marker of myeloid-derived cells, we sought to examine whether BG34-200 could affect specific CD11b^+^ cell subsets in peripheral blood after IV administration. Here, we chose to use a complete separation strategy of CD11b^+^ cells. In the gating strategy, we first gated out Lin (CD3, CD19, CD20, CD56)^+^ cells, divided the Lin^−^CD11b^+^ cells into Ly6G^−^ and Ly6G^+^ cells, and then analyzed the cell expression of CD62L and Ly6C. This allows the phenotypic separation of granulocytic-MDSCs (G-MDSCs) (Lin^−^CD11b^+^Ly6G^+^CD62L^−^Ly6C^−^) and monocytic MDSCs (M-MDSCs) (Lin^−^CD11b^+^Ly6G^−^Ly6C^+^CD62L^−^CCR2^−^). In addition, CCR2 expression by the Lin^−^CD11b^+^Ly6G^−^Ly6C^+^CD62L^+^ allows the phenotypic identification of inflammatory monocytes (Lin^−^CD11b^+^Ly6G^−^Ly6C^+^CD62L^+^CCR2^+^). As compared to PBS control, BG34-200 IV administration resulted in a significant frequency increase in inflammatory monocytes in peripheral blood of mice bearing advanced melanoma (Figure 3A,B). The inflammatory monocytes, Lin^−^CD11b^+^Ly6G^−^Ly6C^+^CD62L^+^CCR2^+^, were not detected in tumor-free mice, suggesting that they were tumor-associated cells (Figure 3B).

To assess whether the tumor-associated inflammatory monocytes (TAIMs) could internalize BG34-200-AF647 after IV administration, we examined the frequency of BG34-200-AF647^+^ cells in the circulating CD11b^+^CCR2^+^ cells and found that more than 30% cells were CD11b^+^CCR2^+^BG34-200-AF647^+^ (Figure 3C). The results of fluorescence microscopic imaging showed that the BG34-200-AF647 molecules were located at the intracellular space of CD11b^+^CCR2^+^ cells, suggesting that the BG34-200-AF647 compound entered the cells (Figure 3D).

It has been shown that, depending on environmental stimuli, functional inflammatory monocytes can differentiate into monocyte-derived macrophages or dendritic cells [28]. Therefore, we sorted TAIMs from the untreated (PBS) and treated (BG34-200) mouse blood and examined the mRNA expression of PU.1 (transcriptional factor regulating cell differentiation into dendritic cells) and MafB (transcriptional factor regulating cell differentiation into macrophages) [28] (Figure 3E). In this experimental setting, we used tumor-free mouse BM-derived inflammatory monocytes as control. BM-derived monocytes were cultured with a low concentration of phorbol 12-myristate-13-acetate (PMA) (20 ng/mL) to polarize cells towards pro-inflammatory activation. Compared with the control group, the PU.1 and MafB mRNA levels of TAIMs in the PBS group were significantly lower, suggesting a relatively poor differentiation ability. In contrast, TAIMs in the BG34-200 group exhibited significantly enhanced PU.1 mRNA expression, suggesting an improved potential for expression of genes necessary for DC differentiation (Figure 3D). Furthermore, we found that these CD11b^+^CCR2^+^BG34-200-AF647^+^ cells displayed no surface expression of MHC II and CD11c (Figure 3F). This is consistent with observations in Figure 2C, suggesting that these circulating monocytes are immature cells as they lack typical DC marker expression.

It has been shown that the tumor stroma produces chemokine CCL2 to increase the release of CCR2^+^ inflammatory monocytes that infiltrate to tumor microenvironment [29,30,31,32,33,34,35]. The CCL2-CCR2 signaling axis association with aggressive tumor progression has been reported in inflammatory breast cancers and pancreatic cancers and demonstrated in the B16F10 model of advanced melanoma. Here, we used this advanced melanoma model to assess whether the CD11b^+^CCR2^+^ cell played a role in the BG34-200-AF647 accumulation in tumor. Our results showed that the signal was observed in B16F10 tumors of WT mice but not in CD11b^−/−^ or CCR2^−/−^ mice (B6.129S4-*Ccr2tm1lfc*/J) (Figure 3G), suggesting that the BG34-200 migration and distribution to tumor tissue was dependent on circulating cells that express inflammatory monocyte markers CD11b and CCR2.

In Figure 2D, the MFI of BG34-200-AF647 in the lungs of CD11b^−^ mice was high at 24 h post-injection. However, in Figure 3G, there appeared to be no obvious fluorescent signals in the lungs of either WT or CD11b^−/−^ mice. This was because the results in Figure 2D were generated using colorimetric quantification, while the results of Figure 3G were generated using non-invasive fluorescence imaging (IVIS spectrum). The IVIS spectrum can well quantify the superficial signals in the subcutaneously injected tumors but was not a good tool to quantify signals in deep tissue such as the lungs. In addition, the scanning was set up to scan the tumor regions, so it was hard to expect detecting signals in the lungs using this IVIS experimental setting.

### 3.4. The Tumor-Infiltrated BG34-200-AF647^+^ Monocytes Can Differentiate into Monocyte-Derived Dendritic cells (mo-DCs) in the Tumor Microenvironment

**Figure 4:**
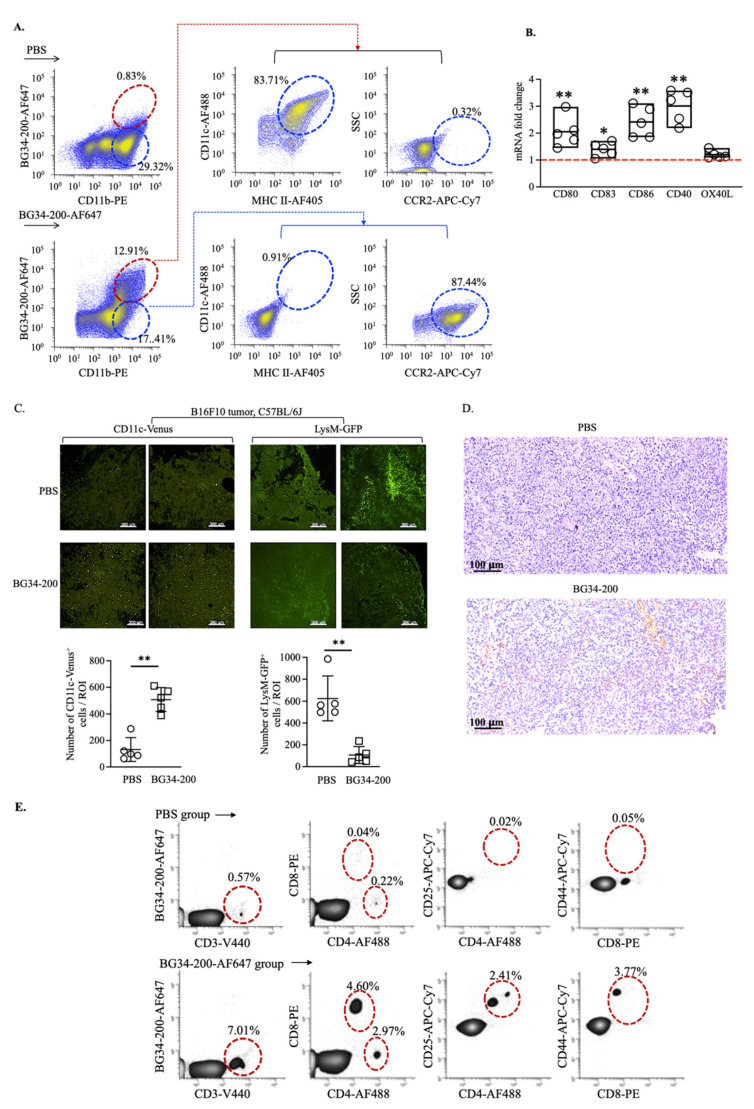
BG34-200 IV administration results in an increase in monocyte-derived DCs in B16F10 tumor microenvironment resulting in the alteration of tumor microenvironment. (**A**). Frequency of tumor-infiltrated CD11b^+^BG34-200-AF647^+^ cells and their expression of MHC II, CD11c, and CCR2, as determined by FACS. (**B**). Fold change of mRNA of CD80, CD83, CD86, CD40, and OX40L in tumor-infiltrating CD11b+BG34-200-AF647^+^ cells. (**C**). In tumors of B16F10 mice receiving PBS or BG34-200-AF647: (left) frequency of tumor-infiltrating CD11c-venus^+^ DC in transgenic CD11c-venus mice; (right) frequency of tumor-infiltrating myeloid-derived monocytes, granulocytes, and mature macrophages in transgenic LysM-GFP mice. (**D**). Tumor-infiltrating T cells (TILs) of B16F10 mice receiving PBS or BG34-200 IV administration, as determined by immunostaining of fixed tumor tissues. Top: Histological section of PBS-treated B16F10 tumors showing no intratumoral TILs. Bottom: Intratumoral TILs in BG34-200-treated B16F10 tumor (brown spots/regions). (**E**). FACS analysis of T-cell population in tumor of B16F10 mice receiving PBS or BG34-200-AF647 IV administration. For (**B**,**C**), *, *p* < 0.05; **, *p* < 0.01.

Here, we studied BG34-200-AF647^+^ cells in tumors at 24 h because the BG34-200-AF647 signal increased to reach peak concentration at ~24 h following IV administration (Figure 2D). Compared with the PBS group, ~10% tumor-infiltrated CD11b^+^BG34-200-AF647^+^ cells were detected in the BG34-200-AF647 group (Figure 4A). The CD11b^+^BG34-200-AF647^+^ cells showed positive expression of CD11c and MHC II and negative expression of CCR2, while the CD11b^+^BG34-200-AF647^−^ cells showed high expression of CCR2 and no expression of CD11C or MHC II (Figure 4A). Compared with CD11b^+^BG34-200-AF647^−^ cells, the CD11b^+^BG34-200-AF647^+^ cells exhibited increased mRNA expression of co-stimulatory factors CD80, CD83, CD86, and CD40 (Figure 4B), which suggested that they were activated DCs.

Further, to assess the change in the TME induced by BG34-200 treatment, we utilized a transgenic CD11c-venus mouse strain (B6.Cg-Tg(Itgax-Venus)1Mnz/J) to examine tumor-infiltrating CD11c^+^ DC frequency (Figure 4C, left). The CD11c-venus mice express yellow fluorescent protein (YFP) under the transcriptional control of the mouse integrin α_x_ (CD11c) promoter, which is exclusively expressed in dendritic cells of the immune system. Our results showed that the BG34-200 treatment resulted in a significant increase in the frequency of DCs in TME (Figure 4C, left). We also utilized a transgenic LysM-GFP mouse strain (B6.129P2-*Lyz2^tm1(cre)Ifo^*/J) to examine the tumor-infiltrating myeloid-derived monocyte, granulocytes, and mature macrophages (Figure 4C, right). Since the LysM-GFP mice have the enhanced green fluorescent protein (GFP) inserted in the lysozyme M (LysM) promoter region, this model provides a method to quantify these cells [36]. These results suggested that the BG34-200 treatment resulted in a reduction in the overall frequency of tumor-infiltrated leukocytes, which may include monocytes, granulocytes, and mature macrophages (Figure, 4C, right). Importantly, we found that BG34-200-induced TME changes were accompanied by increased frequencies of T cells (Figure 4D), which included both CD4^+^ and CD8^+^ T cells expressing activation markers (Figure 4E).

### 3.5. BG34-200-AF647^+^ mo-DCs Migrate to TdLNs, Inducing Melanoma-Specific T-Cell Activation and Expansion

**Figure 5:**
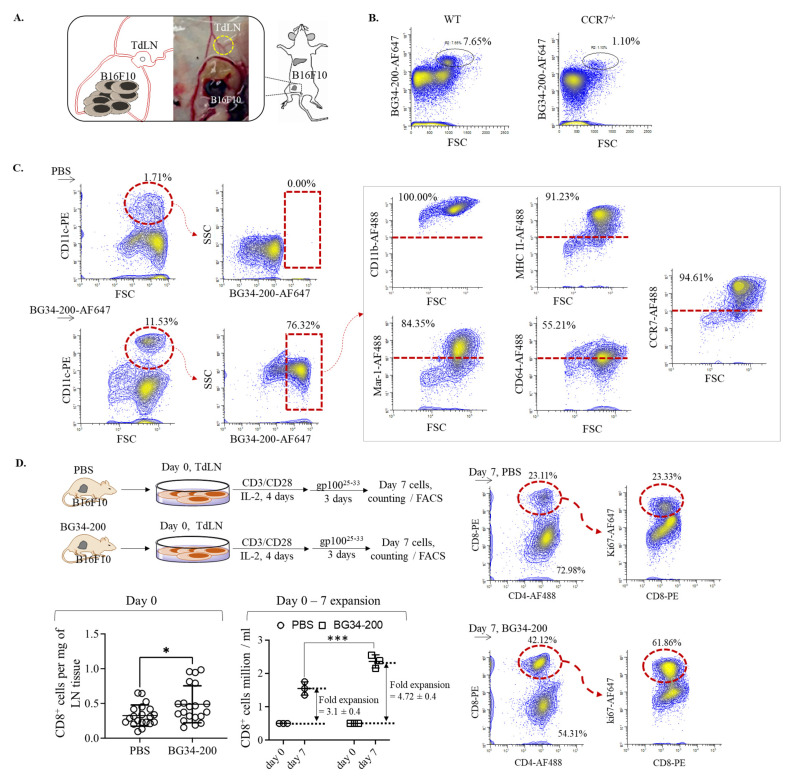
BG34-200-AF647 IV administration influenced cellular migration of mo-DCs and resulted in T-cell priming and expansion in TdLNs. (**A**). Schematics of harvesting TdLNs from B16F10 mice receiving BG34-200 IV administration. (**B**). FACS analysis of fluorescent signal of BG34-200-AF647 in WT and CCR7^−/−^ mice. (**C**). FACS analysis and phenotyping of BG34-200-AF647^+^ cells in TdLNs using PE-tagged CD11c and AF488-tagged CD11b, MHC II, Mar-1, CD64, and CCR7 biomarkers. (**D**). TdLNs harvesting from B16F10 tumor-bearing mice receiving PBS or BG34-200 compound IV injection and culture and expansion of T cells using activation beads in the presence of IL-2 recombinant cytokines. Total number of CD8^+^ cells in TdLNs on day 0 was determined by cell counting of trypan-blue-positive cells and FACS analysis of CD8^+^ cell frequency. The CD8^+^ cell expansion folds were determined by culturing 0.5 million CD8^+^ cells on day 0 and counting the total number of CD8^+^ cells on day 7. The TdLN-derived T-cell proliferation in response to gp100^25−33^ peptides was determined by FACS analysis of the ki67 expression level in CD8^+^ T cells on day 7 cultures. *, *p* < 0.05, ***, *p* < 0.001.

Since we detected BG34-200-AF647 signal in TdLNs at ~72 h after IV administration (Figure 2D), we used this time point to investigate the compound drainage to lymph nodes. To examine it, we utilized WT and CCR7^−/−^ mice (B6.129P2(C)-*Ccr7^tm1Rfor^*/J). The CCR7^−/−^ homozygous mice possess residual lymph nodes that are devoid of migratory DCs, so this mouse strain can be used to differentiate passive lymphatic drainage from cellular drainage [37]. Compared with the PBS group, we found that BG34-200-AF647 signal was significantly decreased in TdLNs of CCR7^−/−^ mice (Figure 5A,B). These results suggest that the infiltration of BG34-200-AF647 to TdLNs is an active cellular transportation rather than passive lymphatic drainage.

### 3.6. BG34-200 IV Administration in Combination with Adoptive Transfer of TdLN-Derived T Cells Induces Significantly Improved Antitumor Effect and Resistance to a Second Tumor Challenge

To examine whether the cellular transport of BG34-200-AF647 to TdLNs was mediated by migratory DCs, we sought to determine the frequency of CD11c^+^AF647^+^ cells in TdLNs. As shown in the results, BG34-200-AF647 IV administration resulted in a significant frequency increase in CD11c^+^ cells, most of which were BG34-200-AF647^+^ (Figure 5 C). Phenotypic analysis of the CD11c^+^BG34-200-AF647^+^ cells showed that these cells exhibited positive expression of CD11b, MHC II, CCR7, Mar-1, and CD64 but negative expression of CD103. CD103 and CCR7 markers were used to identify migratory DCs (CD103^−^CCR7^+^) from resident DCs (CD103^+^CCR7^−^). Mar-1 and CD64 markers were used to identify mo-DCs from conventional DCs (cDCs) because these two markers were reported to perform much better at identifying mo-DCs in lymph nodes than the traditionally used Ly6C markers [38]. This philosophy is very well supported by the presence of cDCs in Flt31^−/−^ mice, showing that the residue lymph node CD11b^+^CD11c^+^ DCs were universally CD64^+^MAR-1^+^ moDCs [38]. Based on this, our results showed that CD11C^+^CD11b^+^BG34-200-AF647^+^ cells in TdLN exhibited a phenotype of migratory mo-DCs because they displayed positive expression of CCR7, Mar-1, and CD64 and negative expression of CD103.

To determine whether the cellular transportation of BG34-200 compounds to TDLNs was accompanied by T-cell activation, we collected TdLNs from PBS- and BG34-200-treated mice, cultured cells in the presence of CD3/CD28 activation beads and recombinant IL-2 and examined the CD8^+^ T-cell proliferation in response to melanoma H2-Db restricted gp100^25−33^ peptide and examined the cell expansion and proliferation (Figure 5D). Compared with the PBS-treated TdLN cultures, BG34-200-treated cultures resulted in ~60% increase in the total number of CD8^+^ cells, as determined by the counting of the total number of trypan-blue-positive cells and the FACS analysis of the CD8^+^ cell frequencies on day 0; the BG34-200 cultures also resulted in an enhanced expansion of CD8^+^ T cells during the 7-day culture. In addition, the CD8^+^ cells of the BG34-200 group showed a significant increase in the expression of proliferation maker Ki67. Collectively, these results suggested that the BG34-200 treatment could induce melanoma specific CD8^+^ T-cell expansion through promoting cell proliferation (Figure 5D).

**Figure 6:**
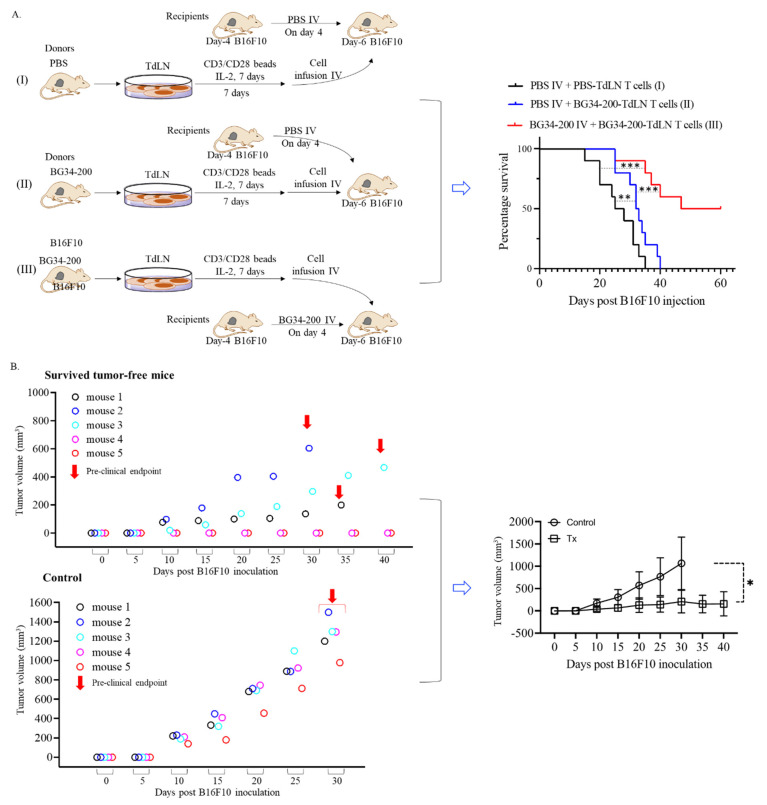
TdLN-derived T cells from BG34-200-treated donor B16F10 mice induce significantly improved antitumor effect in recipient mice bearing advanced B16F10 tumors. (**A**). Kaplan–Meier overall survival curves of recipient mice in group I, II and III. The TdLNs were harvested from donor B16F10 mice receiving a PBS (group I) or BG34-200 (group II and III) intravenous injection, cultured/expanded using T-cell activation beads (CD3/CD28) in the presence of recombinant IL2 cytokines, and adoptively transferred to recipient mice. For the donor mice, one million B16F10 cells were injected into left flanks on day 0; PBS (group I) or BG34-200 (groups II and III) were intravenously administrated on day 4. For the recipient mice, 1.5 million B16F10 cells were injected into left flanks on day 0; PBS (group I and II) or BG34-200 (group III) were intravenously administrated on day 4; adoptive transfer of T cells was conducted on day 6. (**B**)**.** Tumor volume of surviving mice after a second inoculation of B16F10 cells. The surviving mice referred to the five tumor-free mice in group III and received a second tumor cell inoculation. Five naïve tumor-free mice served as control. All the mice were inoculated with 0.25 million tumor cells into the right flank and tumor growth was monitored. *n* = 5. For A and B, *, *p* < 0.05; **, *p* < 0.01; ***, *p* < 0.001.

The BG34-200-induced T-cell activation in TdLNs inspired us to evaluate a therapy combining BG34-200 IV administration and the adoptive transfer of TdLN-derived T cells. Kaplan–Meier curves demonstrated that the combination therapy improved the animal survival, and 5 of 10 mice of the BG34-200 group were found to be tumor free (Figure 6A). These results suggested that the BG34-200 and T-cell combination therapy resulted in a significantly improved efficacy and good response rate. Importantly, after the five tumor-free mice received a second subcutaneous inoculation of B16F10 tumor cells, two mice remained tumor free for 40 days, and the other three showed delayed tumor growth (Figure 6B, left), as compared to the tumor-free control mice (Figure 6B, right). Since the combination therapy experiment involved the use of donor/recipient mice and adoptive cellular transfer and took a long time to maintain the tumor free mice, the five naïve mice in the control group in Figure 6B were not age matched. The control mice were naïve animals that did not receive any previous treatment or tumor cell inoculation. However, there was a significant difference between these two groups at *n* = 5. These results suggested that BG34-200 IV treatment combined with T-cell adoptive transfer could significantly enhance the antitumor effect of BG34-200 alone, and 40% of the surviving animals showed resistance to a second tumor cell challenge.

## 4. Discussion

Modulating immune responses using β-glucans has been investigated for decades in solid tumor cancers. The majority of human clinical experience has been the use of fungi- or yeast-derived β-glucan in combination with tumor-targeting antibodies, tumor vaccines or immune checkpoint inhibitors (e.g., NCT01829373, NCT00682032, NCT00289003, NCT00492167, NCT00089258, and NCT02981303). Some of these β-glucans display the capability of modulating innate immune responses via interacting with toll-like receptors (TLRs) [39,40,41]. Our results suggest that the β-glucans ligand BG34-200 can modulate immune responses via interacting with a C-type integrin CD11b, which shows promising antitumor effects in a mouse model of advanced melanoma (Figure 1).

Our data support a model that the direct entry of BG34-200-AF647 to circulating CD11b^+^ cells facilitated the plasma clearance of the compound and showed an impact on the cell migration and differentiation in the context of advanced melanoma (Figure 2 and Figure 3). BG34-200 exposure in the peripheral blood appeared to result in a significant increase in PU.1 expression in the tumor-associated inflammatory monocytes (CD11b^high^Ly6C^high^CCR2^+^PU.1^low^). Although the high level of PU.1 expression versus MafB often suggests monocyte differentiation towards DCs, we found that these circulating CD11b^high^Ly6C^high^CCR2^+^PU.1^high^ subsets displayed negative surface expression of MHC II and CD11c (Figure 3B–F), suggesting that they were not differentiated or matured DCs.

It has been shown that the microorganism-derived substances (such as virus-, bacteria-, fungi-derived cell wall peptide, protein, polysaccharides, or complexes) can trigger the differentiation of inflammatory monocytes into microbicidal macrophages or mo-DCs [42]. However, it is unclear whether environmental inflammatory cues control the polarization of monocytes towards each of these fates. Our data suggest that BG34-200, the plant-derived cell wall polysaccharide, may function as an external microbial stimulus that effectively potentiates the tumor-associated inflammatory monocytes (CD11b^high^Ly6C^high^CCR2^+^PU.1^low^) into the CD11b^high^Ly6C^high^CCR2^+^PU.1^high^ subsets in circulation. These CD11b^high^Ly6C^high^CCR2^+^PU.1^high^ subsets could migrate to TME (Figure 3G), where they lose their surface expression of CCR2 and exhibit positive expression of DC biomarkers MHC II and CD11c (Figure 4A). These results suggest that BG34-200 exposure results in the potentiation of TAIMs in peripheral blood, which enhances cell differentiation into mo-DCs in melanoma tumors.

In B16F10 tumors, we have previously identified upregulation of several proinflammatory cytokines (GM-CSF, TNF-α, IFN-γ, and IL-4), which was thought to be induced by BG34-200-mediated downregulation of MDSCs and M2 macrophages and upregulation of TILs [8]. This is consistent with and well supported by this current study (Figure 4C–E). Although these factors are beneficial for creating an immunogenic tumor microenvironment, it is unclear whether they are sufficient to support mo-DCs differentiation. On the other hand, we have previously observed that the BG34-200 treatment could trigger CD11b-mediated internalization of the binding complexes by inflammatory monocytes in the context of solid cancers. This resulted in enhanced phagocytosis and F-actin cytoskeletal rearrangement, which appeared to be very important structural biological change essential for mo-DC differentiation. In addition, our results show that the Ca^2+^ binding to the CD11b I domain overlaps with the BG34-200-CD11b binding epitopes. Together, these results imply that BG34-200 engagement with integrin CD11b may induce direct change in metabolic networks important for mo-DC differentiation, which is being investigated in our ongoing study.

**Figure 7:**
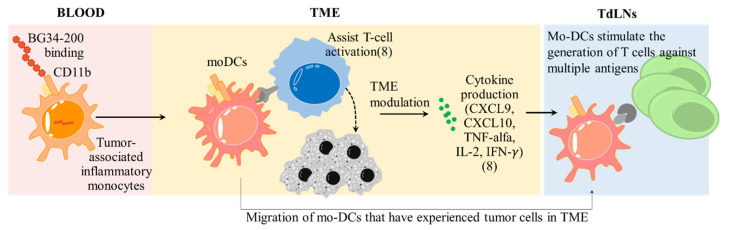
Sequential immune activation induced by BG34-200 IV administration in the context of advanced melanoma. In peripheral blood, BG34-200 IV administration triggers direct entry of compound to the tumor-associated inflammatory monocytes (CD11b^+^CCR2^+^PU.1^low^) and potentiates the cells to be CD11b^+^CCR2^+^PU.1^high^. The BG34-200^+^CD11b^+^CCR2^+^PU.1^high^ cells migrate to TME and differentiated into mo-DCs, resulting in alteration of tumor microenvironment, as evidenced by ref. [8] and this current study. The mo-DCs are found to migrate to TdLNs, where they stimulate the generation of tumor-specific T cells. The BG34-200-induced antitumor effect is unique in that the source of tumor-specific T cells is derived from the sentinel draining lymph nodes, which is the natural site of the immune response against pathogens as well as cancer. This can potentially improve the T cells that are naturally primed against defined and undefined tumor antigens, leading to a broader response to tumor antigens.

Furthermore, we found that the transport of fluorescent BG34-200 molecules to TdLNs occurred through active cellular transport and not via passive lymphatic drainage because the accumulation of fluorescent BG34-200 was significantly decreased in the TdLNs of CCR7^−/−^ mice (Figure 4A,B). Most of the BG34-200-AF647 signal was found in CD11b^+^CD11c^+^ cells that are MHCII^+^CCR7^+^, suggesting that they were migratory DCs (Figure 4C). Since the CD11b^+^CD11c^+^BG34-200-AF647^+^ cells were found in tumors (Figure 3A), these results suggest that these cells might be migratory mo-DCs that were transported from the tumor site to the sentinel draining lymph nodes and resulted in the proliferation of melanoma-specific CD8^+^ T cells in TdLNs (Figure 5D). Collectively, these results suggest that BG34-200 IV administration showed an impact on the tumor-associated inflammatory monocytes that effectively induced sequential immune activation in the context of advanced melanoma (Figure 7).

Importantly, our results suggested that the BG34-200 treatment could improve systemic T cell priming because TdLN-derived CD8^+^ T cell frequency was higher on day 0 and melanoma-specific T cells were more proliferative on day 7 (Figure 5D). T-cell priming in sentinel draining lymph nodes occurred naturally in vivo by the progressively growing melanoma, and TdLN-derived T-cell therapy has been tested in early clinical trials of stage III–IV melanoma. Our finding is that BG34-200 results in systemic T-cell priming in vivo. This is different from ex vivo priming with antigen or in vivo vaccination. This can potentially improve T cells that were naturally primed in vivo against defined and undefined tumor antigens, which may lead to a broader response to tumor antigens. In a previous phase I clinical trial, we cultured and expanded tumor-specific T cells from melanoma-draining regional lymph nodes in patients who had undergone lymph node dissection. This study is unique in that the source of T cells for therapy is derived from the lymph node, which is the natural site of the immune response against pathogens as well as cancer. This phase I trial of stage III melanoma has demonstrated that the infusion of activated T cells is an area of great potential for the treatment of patients with advanced melanoma, and the method of activating and expanding TdLN T cells is practical and may be ideal for widespread use. Here, our results showed promising efficacy combining BG34-200 IV administration with the adoptive transfer T-cell therapy (Figure 6).

Melanoma is the fastest increasing tumor worldwide. Immunotherapy using immune checkpoint inhibitors (e.g., PD-1) is now the first-line standard of care, but > 50% of patients still do not benefit (even from combined use of the inhibitors) [43,44]. Now there are no good treatment options after failure of PD-1. The scientific findings from this study will allow us to develop new BG34-200 and T-cell combination therapy, which will benefit patients with advanced melanoma who received standard of care treatment with and without immunotherapy and may result in cures.

## 5. Conclusions

Immunotherapy using immune checkpoint inhibitors is now the first line standard of care for advanced melanoma but >50% of patients still do not benefit. Using our recently developed plant-derived molecule we call BG34-200, we found that BG34-200 IV administration could significantly inhibit tumor growth and improve survival in B16F10 mice with advanced melanoma. Our data supported that BG34-200 could target elements of myeloid components (i.e., tumor-associated inflammatory monocytes), to improve systemic T cell priming in both tumor microenvironment and TdLNs. Based upon these results, we combined BG34-200 IV administration with adaptive transfer of TdLN-derived T cells, which significantly improved animal survival and helped tumor-free mouse survivors be resistant to a second tumor-cell challenge. The scientific findings of this study suggest that the BG34-200-based combination immunotherapy can be developed to benefit patients with advanced melanoma who do not respond to current standard of care therapies with and without immunotherapy.

## Data Availability

All data generated or analyzed during this study are included in this article.
